# The Rhinoplasty Outcome Evaluation (ROE) Questionnaire in Rhinoplasty: A Systematic Review and Meta-Analysis

**DOI:** 10.3390/jcm13164642

**Published:** 2024-08-08

**Authors:** Piotr Rot, Sandra Krzywdzińska, Paweł Grab, Dariusz Jurkiewicz, Aldona Chloupek, Maria Sobol

**Affiliations:** 1 Clinic of Otolaryngology and Oncological Otolaryngology with Clinical Department of Cranio-Maxillofacial Surgery, Military Institute of Medicine-National Research Institute, 04-141 Warszawa, Poland; rot@autograf.pl (P.R.);; 2 Department of Cranio-Maxillofacial Surgery, Military Institute of Medicine-National Research Institute, 04-141 Warszawa, Poland; 3 Department of Biophysics, Physiology and Pathophysiology, Medical University of Warsaw, 02-091 Warszawa, Poland

**Keywords:** ROE, the Rhinoplasty Outcome Evaluation, meta-analysis, quality of life

## Abstract

**Background/Objectives:** This study aims to systematize the ability to use ROE to assess rhinoplasty outcomes in surgical approaches. **Methods:** The PubMed, Scopus, and Web of Science databases were searched for the following terms: “rhinoplasty and outcome” OR “prognosis” OR “outcomes” OR “satisfaction” OR “quality of life” OR QoL “rhinoplasty outcome evaluation”. The timeframe of the included studies is from 2011 to May 2024. Ultimately, 17 papers were included in the conducted meta-analysis of ROE scores between pre- and post-treatment data. **Results** The mean value of the pre-treatment ROE score was 33.50 with a CI of 29.46 to 37.53 (*p* < 0.001), while the post-treatment ROE was 69.60 with a CI of 63.07 to 76.14 (t ≤ 6 months). At t = 12 months it was 80.25 with a CI of 75.79 to 84.70 (*p* < 0.001). The mean difference between pre-treatment and post-treatment scores (t ≤ 6 months) was −36.31 with a CI of −40.93 to −31.69. The mean difference between pre-treatment and post-treatment scores for 6 m < t ≤ 12 m was −47.36 with a CI of −53.89 to −40.83. **Conclusions:** The result was statistically significant (*p* < 0.001).

## 1. Introduction

The nose is a distinctive facial feature of great aesthetic importance. As the central landmark of the face, its distance from other anatomical structures, dimensions, and symmetry directly affect the beauty of the face. It is crucial for the individual identity of any person. Among others, overall facial harmony and aesthetics hugely depends on the symmetry and spatial relations of the nose.

Rhinoplasty is one of the most common procedures performed in the plastic surgery/otolaryngology medical fields. The main indications are aesthetics and nasal obstruction [1]. According to the literature, rhinoplasty patients are less satisfied with the achieved results in comparison with those who underwent other facial plastic surgery procedures. Due to its aesthetic nature, having a great impact on the self-image and self-esteem of the patient, the assessment of the final rhinoplasty results should be made from the patients’ viewpoint [2]. Imposing criteria that determine aesthetics is not an easy task. For this reason, numerous questionnaires are available to evaluate quality-of-life change after the procedure (e.g., Rhinoplasty Outcome Evaluation, Sino-Nasal Outcome Test, Nasal Obstruction and Septoplasty Effectiveness Scale, and Standardized Cosmesis and Health Nasal Outcomes Survey) [3,4].

The ROE (Rhinoplasty Outcome Evaluation) is an easy-to-use questionnaire developed to assess the satisfaction and final results of rhinoplasty. It consists of six questions, two for each factor regarding the physical, emotional, and social dimensions of patient satisfaction. The author of the questionnaire took into account the main factors affecting a patient’s satisfaction with the surgery: physical, which examines the patient’s satisfaction with the shape and function of the nose; emotional, which assesses the level of self-confidence and willingness to change appearance; and the social factor, which assesses social, professional, and family acceptance. Since its creation, it has been translated into many languages and popularized as an evaluation method [5,6].

In this meta-analysis, we aim to systematize the ability of the ROE to assess rhinoplasty outcomes in surgical approaches.

## 2. Materials and Methods

**Chart 1:**
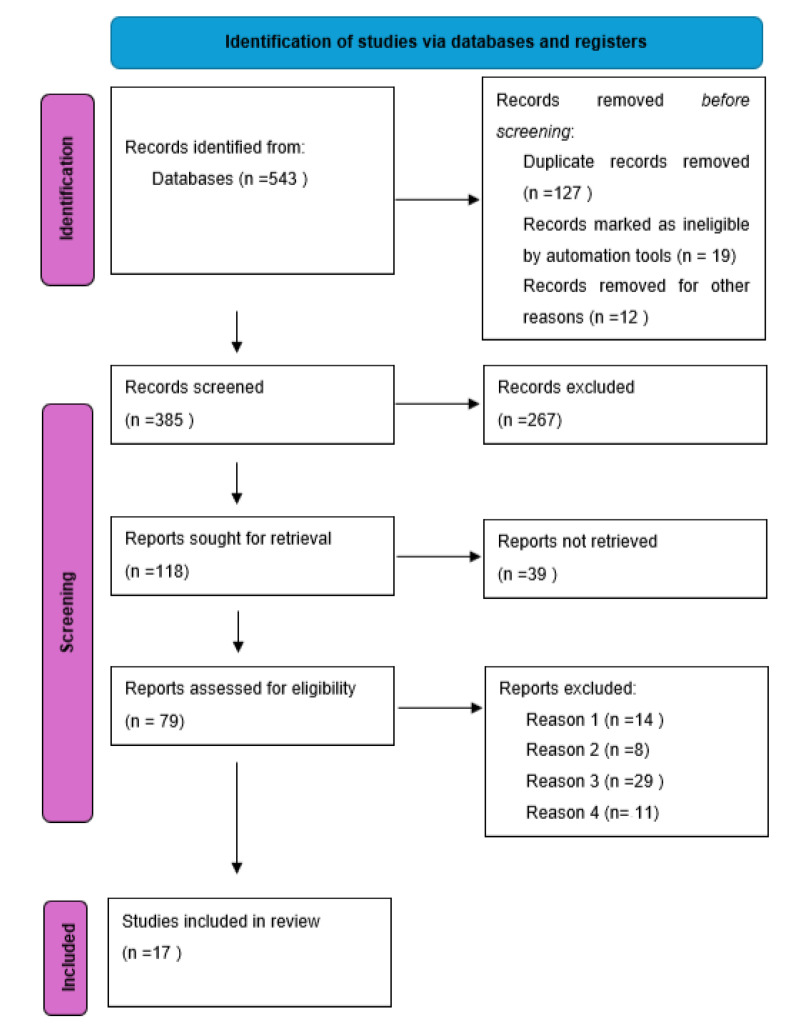
Flow chart for inclusion of articles.

Studies included in this research were selected during a systematic search of literature in databases such as Scopus, Web of Science, and PubMed. The pool of studies was narrowed down to papers published from January 2017 to July 2023. The screening of the results was based on the phrases: “rhinoplasty and outcome” OR “prognosis” OR “outcomes” OR “satisfaction” OR “quality of life” OR QoL “rhinoplasty outcome evaluation”. The search was performed in abstracts and full texts. Two reviewers, the first and the third authors of this article, assessed each abstract and full text for potential inclusion and reached a consensus for the articles to be included in the final review. The articles were excluded if they: (1) were written in any language other than English, (2) did not evaluate the patients’ satisfaction and quality of life or (3) used a scale different than the Rhinoplasty Outcome Evaluation (ROE) for the evaluation of the quality of life (QoL) and patients’ satisfaction, or (4) did not report results as the mean and standard deviation. Seventeen articles were taken into account after repetitive papers were eliminated [7,8,9,10,11,12,13,14,15,16,17,18]. (Chart 1).

### 2.1. Study Selection

The systematic review was conducted using the PRISMA guidelines [19]. The detailed requirements are listed below:

- Inclusion criteria: original, prospective, or retrospective articles concerning rhinoplasty with ROE scores, human subject studies published in English, adults, and studies published up to and inclusive of July 2023;
- Exclusion criteria: article types such as case reports, editorials, letters, books, and conference papers; papers in which the results were presented as the mean without the standard deviation, or the median and range, or the median range and interquartile range; or studies with the ROE expressed as graphs, without values.

### 2.2. Data Extraction

The following data were extracted from each study: the authors, year of publication, number of participants, sex of participants, entry age, Rhinoplasty Outcome Evaluation scores before and after rhinoplasty, the duration of follow-up, presenting deformity, technique used, and the study site.

### 2.3. Statistical Analysis

**Figure 1:**
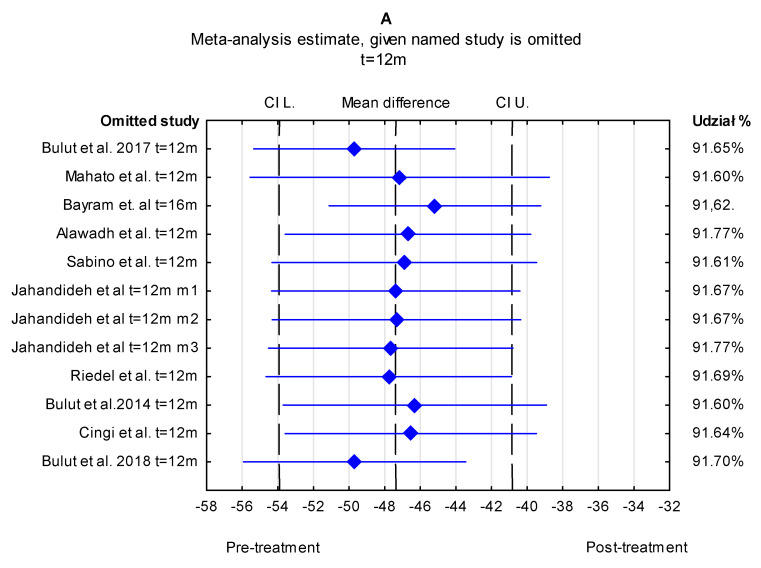

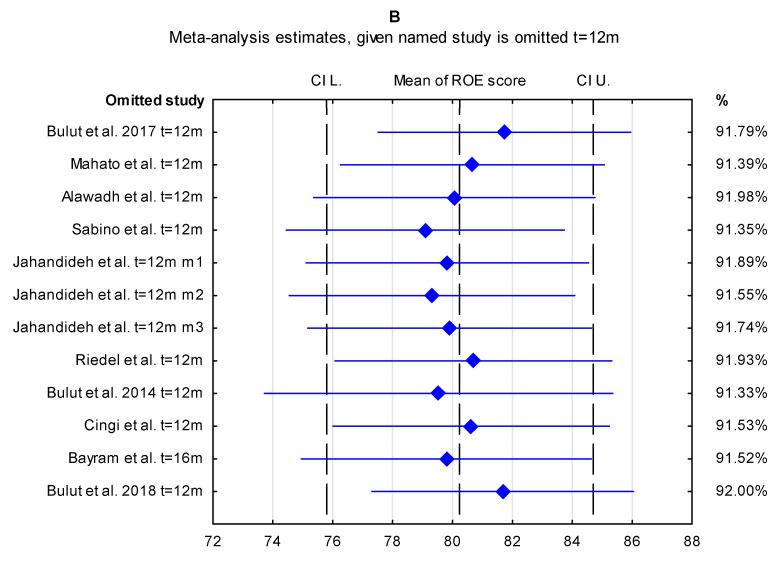
Sensitivity analysis for the effect of individual studies (given named study in the Y axis is omitted) on the pooled differences of ROE score. CI (confidence interval). (**A**) pre-treatment vs. post-treatment t = 12 m, (**B**) mean value of ROE score t = 12 m.

Statistical analysis was conducted using Statistics 13 (Dell Software Inc., StatSoft Warsaw, Poland). The Q test was used to test heterogeneity, and the I2 statistic was carried out to quantify and evaluate heterogeneity. The I2 values ranged from 0 to 100% with higher values indicating greater heterogeneity (25–50%—low, 50–75%—moderate, and >75%—high). Since in all the conducted analysis, heterogeneity scores exceeded 75%, the analysis was performed using a random effects model, and the standardized mean difference was provided with 95% confidence intervals (95% CI). The results were as follows: 99.14% (95% CI of 99.00% to 99.26%) in pre-treatment ROE scores, 99.51% (95% CI of 99.42% to 99.59%) in pre-treatment vs. post-treatment t ≤ 6 months, 99.58% (95% CI of 99.50% to 99.64%) in pre-treatment vs. post-treatment 6 m < t ≤ 12 m, 99.50% (95% CI of 99.40% to 99.58%) in post-treatment t ≤ 6 months, and 99.53% (95% CI of 99.44% to 99.60%) in post-treatment 6 months < t ≤ 12 months. Forest plots were generated to describe the change in Rhinoplasty Outcome Evaluation scores before and after rhinoplasty based on the duration of follow-up (≤6 months, and ≥12 months). To assess publication bias, Egger’s test and Begg’s test were performed. To assess the stability of the results, sensitivity analysis was conducted by excluding each study at a time (Figure 1). Moreover, the trim-and-fill method for publication bias was performed to estimate potentially missing studies.

## 3. Results

**Figure 2:**
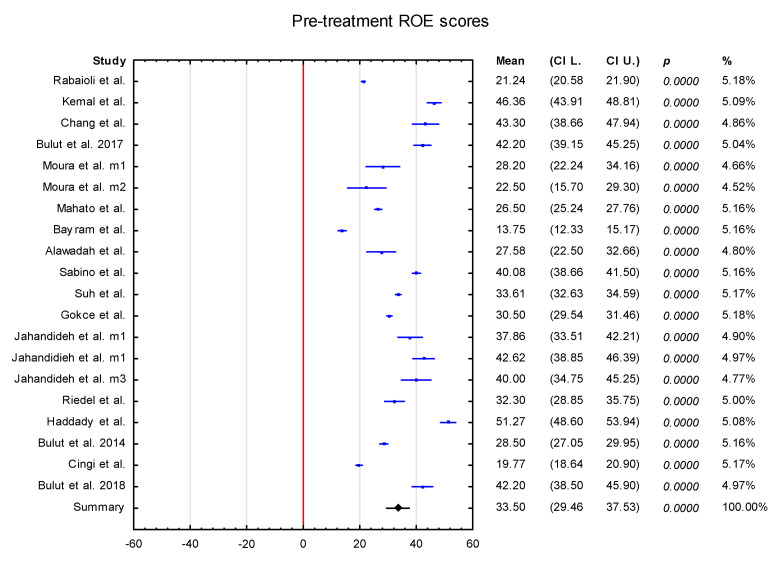
The pre-treatment ROE scores.

As regards the eleven included papers, the mean pre-treatment ROE score was 33.50 with a CI of 29.46 to 37.53 (Figure 2). The result was statistically significant.

**Figure 3:**
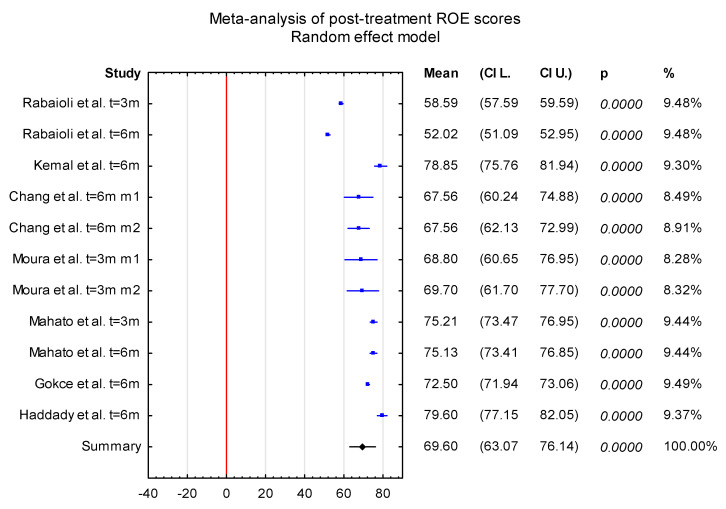
The post-treatment ROE scores (t ≤ 6 months).

The mean post-treatment ROE score (t ≤ 6 months) was 69.60 with a CI of 63.074 to 76.14. The result was statistically significant (Figure 3).

**Figure 4:**
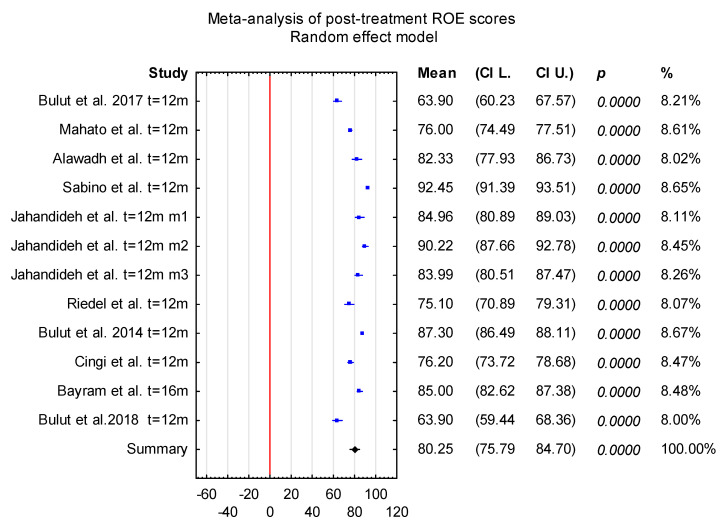
The post-treatment ROE scores (t = 12 months).

The mean post-treatment ROE score (t = 12 months) was 80.25 with a CI of 75.79 to 84.70. The result was statistically significant (Figure 4).

**Figure 5:**
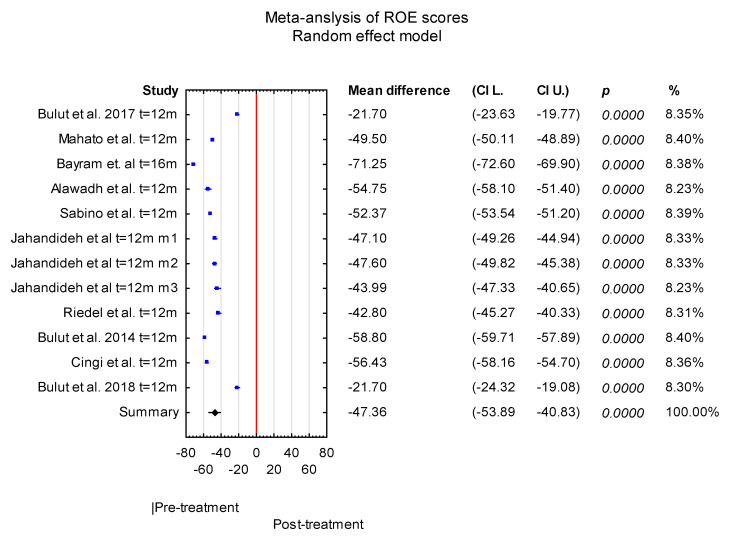
The difference between pre-treatment and post-treatment scores 6 months < t ≤ 12 months.

The mean difference between pre-treatment and post-treatment scores (6 months < t ≤ 12 months) was −47.36 with a CI of −53.89 to −40.83. The result was statistically significant (*p* < 0.001) (Figure 5).

**Figure 6:**
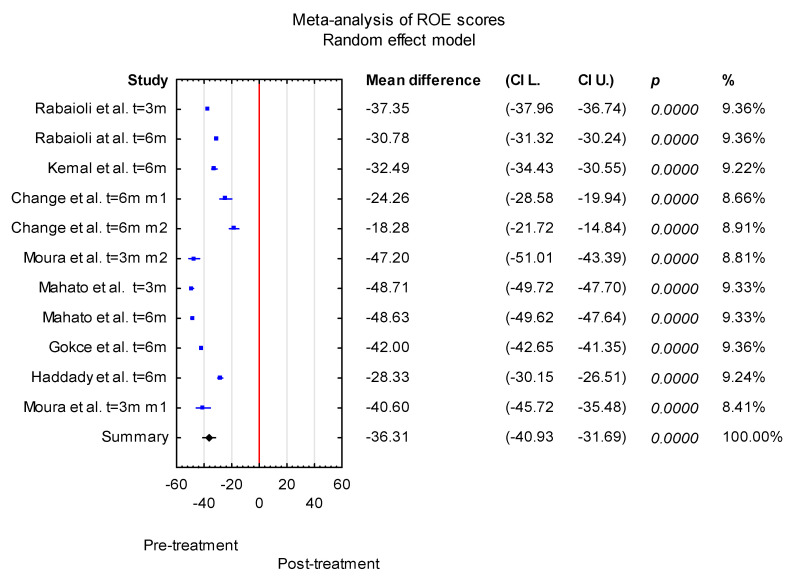
The difference between pre-treatment and post-treatment scores for 0 m < t ≤ 6 m.

The mean difference between pre-treatment and post-treatment scores for 0 m < t ≤ 6 m was −36.31 with a CI of −40.93 to −31.69. The result was statistically significant (*p* < 0.001) (Figure 6).

Overall, the forest plot for the pre-treatment vs. post-treatment results revealed that the studies denoted a higher value of ROE scores after treatment, and the increase was statistically significant in each case. However, there were differences among the included studies.

An Egger’s publication bias was generated. The results of Egger’s test (*p* = 0.535 pre-treatment, *p* = 0.123 post-treatment t = 3 m and 6 m, *p* = 0.068 post-treatment t = 12, and *p* = 0.367 pre-treatment vs. post-treatment t = 12 m) indicated a minimal potential risk of publication bias, which was also consistent with the Begg’s test results.

### 3.1. The Subgroup Analysis

**Figure 7:**
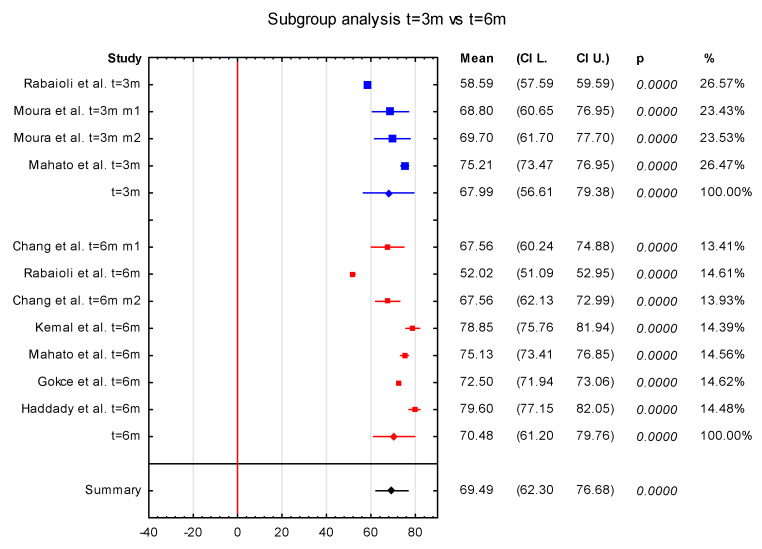
The subgroup analysis for follow-up time.

**Figure 8:**
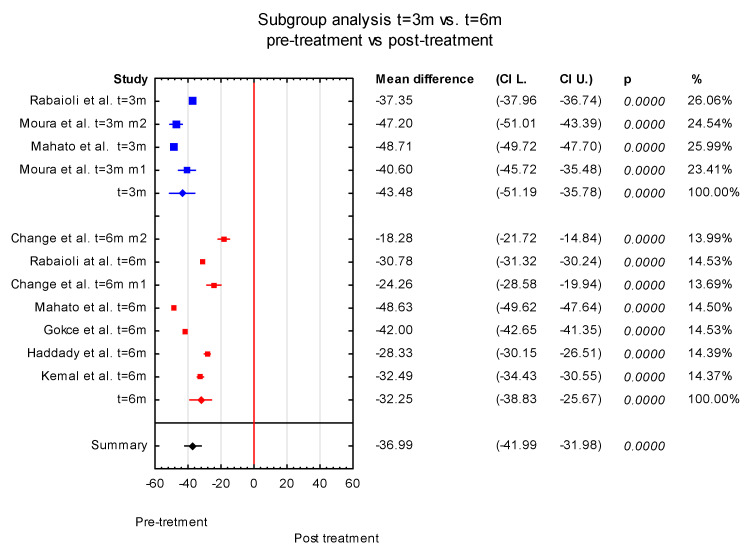
The subgroup analysis of difference between pre-treatment and post-treatment (3 months and 6 months).

**Figure 9:**
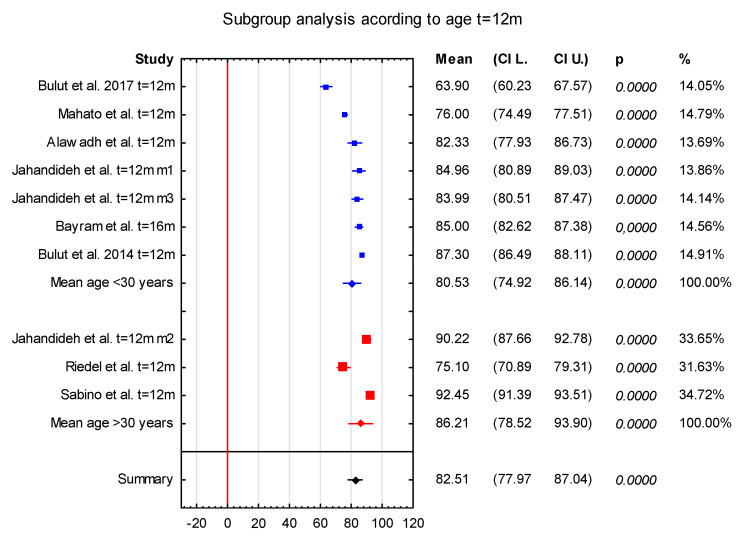
The subgroup analysis t = 12 m according to age groups.

**Figure 10:**
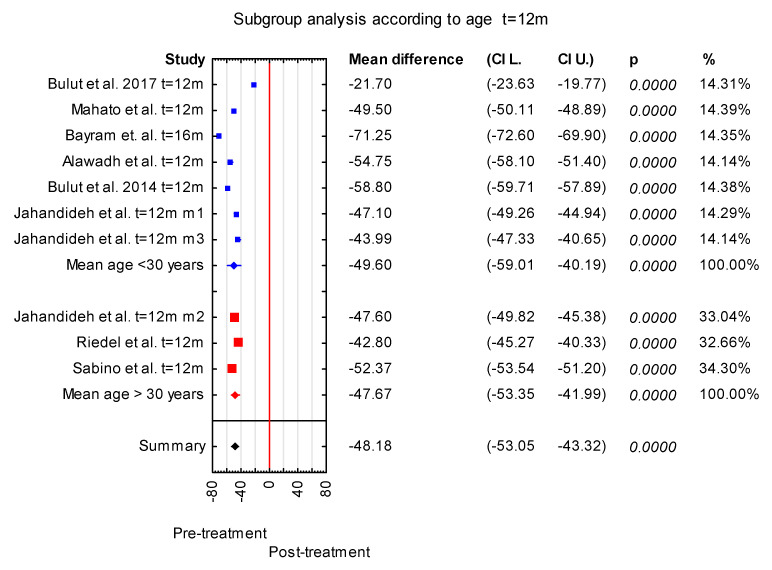
The subgroup analysis of difference between pre-treatment and post-t = 12 m according to age group.

**Figure 11:**
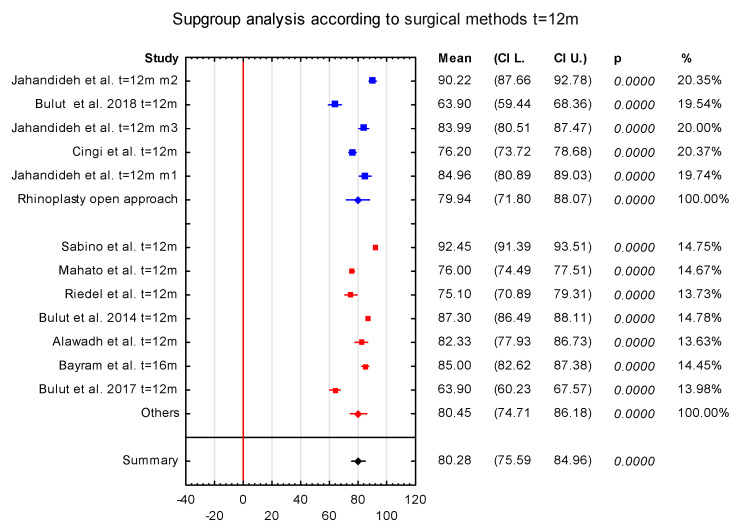
The subgroup analysis t = 12 m according to surgical methods.

Since the heterogeneity among studies was high, a subgroup analysis was performed to try to find possible reasons for the variability of the results. The different time of grading of postoperative follow-up processes (3 months or 6 months), age (age < 30 years and age > 30 years), and surgical methods (rhinoplasty open approach vs. others) for time t = 12 m were considered for the subgroup analysis. In four publications that were included in the meta-analysis, results of the ROE were given 3 months after the procedure, and in seven publications the results were given after 6 months. Statistically significant results were received for the two subgroups (Figure 7 and Figure 8). Moreover, to conduct subgroup analysis for t = 12 m according to age, the considered publications were divided into two groups, patients with mean age lower than 30 years (group 1) and mean age higher than 30 years (group 2). For this division, the papers by Cingi et al. and Bulut et al. were excluded, as the mean age of the group of patients was not given [20,21]. In seven publications, the mean age of the group of patients was lower than 30 years, and in three it was higher than 30 years. Statistically significant results were received for the two subgroups (Figure 9 and Figure 10). Then, papers were divided according to surgical methods; five publications included open-approach rhinoplasty, while in the other eight involved other techniques. Statistically significant results were received for the two subgroups (Figure 11). However, it is important to note that for each case of division one of the subgroup analyses contained only three studies; thus, the true effect is difficult to establish.

The subgroup analysis was also performed in case of mean difference between pre-treatment and post-treatment (3 months and 6 months). Here, we also received statistically significant results for the two subgroups (Figure 8).

### 3.2. The Trim-and-Fill Method

**Figure 12:**
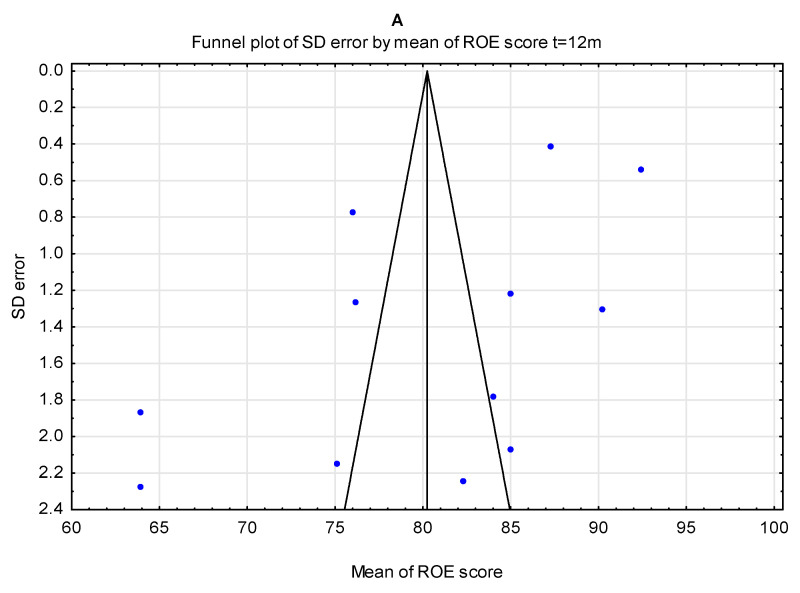

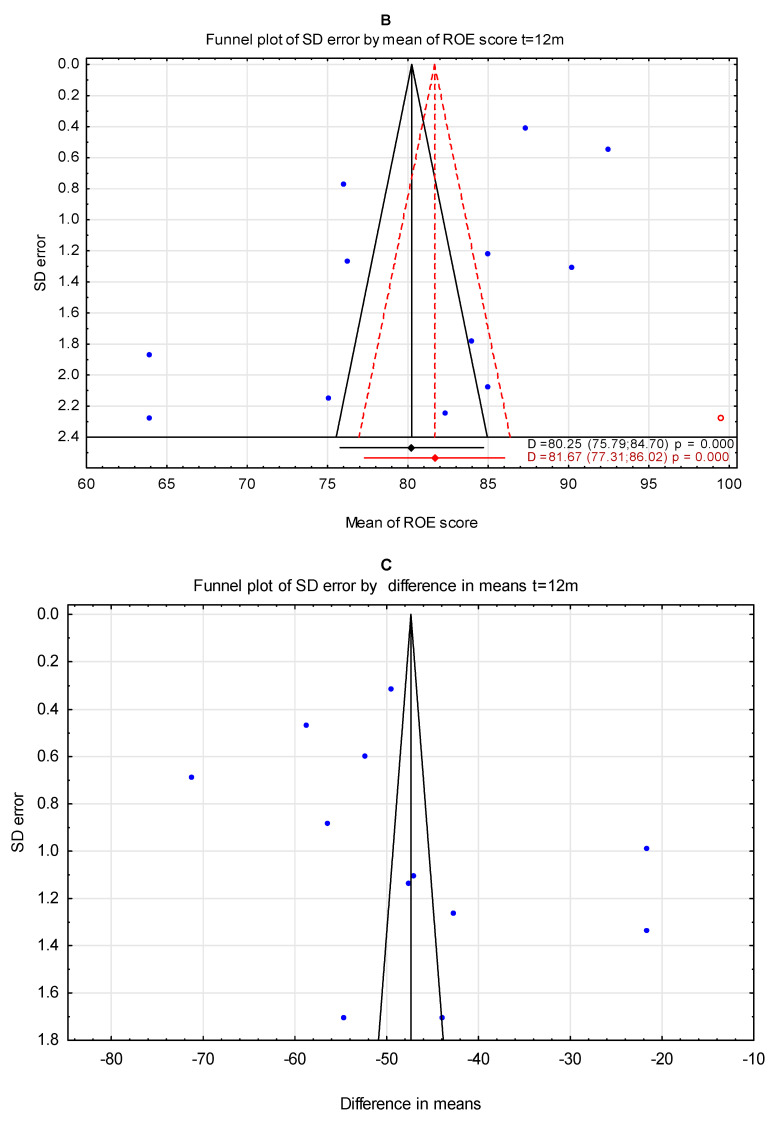

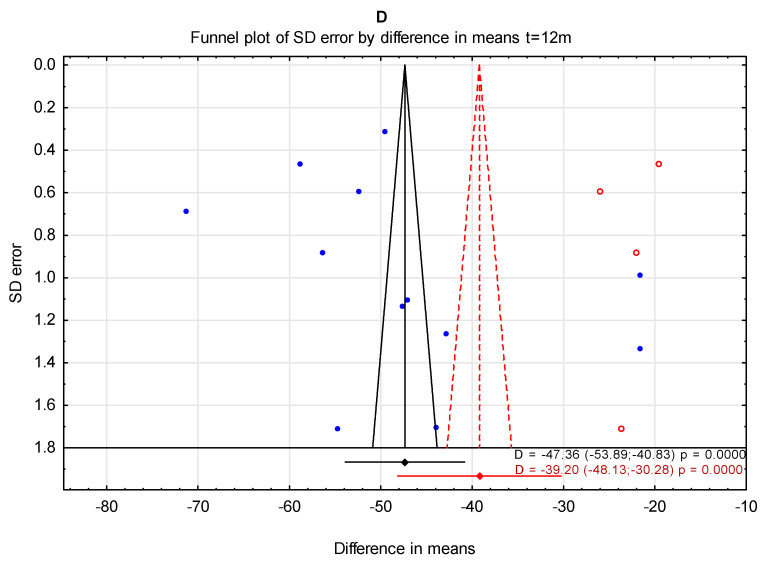
Funnel plots before (**A**,**C**) and after (**B**,**D**) applying the trim-and-fill method; imputed missing studies (red dots) t = 12 m.

The trim-and-fill method was used to estimate the effect of potentially missing studies due to publication bias in the funnel plot for mean post-treatment ROE score and mean difference pre-treatment vs. post-treatment ROE score after t = 12 m. In the case of mean post-treatment ROE score, the received value was 81.67 (95% confidence interval from 77.31 to 86.02), and for pre-treatment vs. post-treatment the value was −39.20 (95% confidence interval from −48.13 to −30.26) (Figure 12).

## 4. Discussion

Nasal physiology is a complex subject. The nose, as a complex structure, is a sensory organ participating in breathing as well as olfaction. Both of these functions are reliant on nasal airflow, which must be considered during mid-face surgical procedures [2,22]. Therefore, disorders of the nose affect not only its external appearance but also the inside of the nose, which may pose a real challenge for surgeons.

Rhinoplasty is a procedure whose aim is to address two aspects: achieving aesthetic goals with the simultaneous maintenance or improvement of nasal airway function. It is a complex surgery, so it has to involve a careful evaluation of overall facial symmetry, bones, and cartilage that create unique anatomy; it must consider issues such as external nasal deformity, septal deviation, turbinate hypertrophy, and nasal tip ptosis. Many patients with nasal deviation are unaware of any inherent facial asymmetries. Recognizing and discussing the interrelation between the deviated nose and asymmetry is an important factor taken into consideration before surgery [1]. The knowledge and understanding of nasal anatomy are therefore necessary for successful nasal reconstruction [22].

Rhinoplasty is one of the most complex surgeries that has been developing globally in the last decade. For example, over 200,000 nose reshaping surgeries were performed in the United States in 2018 [23,24]. Anco et al., in the study Preservation Rhinoplasty: A New Approach to Mestizo Noses, state that rhinoplasty is the fourth most requested procedure worldwide [25]. As mentioned above, the aim of a well-received nasal reconstruction is to achieve aesthetic goals while maintaining or improving nasal airway function. Rhinoseptoplasty has changed significantly since the first aesthetic nose surgery was performed. A great variety of surgical techniques and approaches are available [26]. In the pursuit of better and more consistent results, the reduction-only concept in nasal surgery has gradually been replaced by a more sparing approach. Surgeons focus on careful, minimal reduction and reimplantation of collected materials. After assessing the results of the procedures performed, the emphasis is currently placed on short-and long-term outcomes, possible complications, and secondary treatment options. Therefore, it is believed rhinoplasty could have an impact on quality of life [22].

It is worth noting that rhinoplasty, like any other surgical procedure, is associated with the possibility of occurrence of complications and the risk that there may be a discrepancy between the expectations of the patient and the doctor. For this reason, it seems crucial to conduct a properly performed initial examination, determine the patient’s expectations, and establish achievable goals for the surgery, including educating the patient about possible complications.

Complications that may occur include pain, swelling, infection, bleeding in the early and late postoperative period, postoperative deformities, discoloration of the incisors, scars, and breathing disorders, including the feeling of a blocked nose. Infections are extremely rare but can lead to a life-threatening condition if toxic shock syndrome develops. The risk is greater when sinus surgery and rhinoplasty are performed at the same time.

Skin and soft tissue complications include transient side effects such as postoperative swelling and hematomas. Late complications that are important for the patient include atrophy, fibrosis, the formation of unsightly scars, a feeling of numbness/stiffness, or even the formation of cysts from displaced mucosa or subcutaneous granulomas.

Postoperative deformities are considered the main risk of rhinoplasty and are the reason for reoperation in up to 5–15% of patients, depending on the sources. According to data, one of the most common postoperative deformities is “pollybeak”. The risk of this complication increases when, before surgery, the patient has a deep nasofrontal angle, a cartilaginous hump, and reduced apical projection. Pollybeak is the cause of up to 50% of all reoperations. Other common postoperative deformities include a drooping and wide nasal tip, recessed nasal column base, or nasal bridge irregularities [27,28].

The risk of complications can be reduced with increasing surgeon experience and previously established patient expectations. It needs to be underlined that we do not have good statistics describing frequency or percentage of possible complications, which should be a topic for further investigation.

Despite the common interpretation of assigning an aesthetic character to rhinoplasty, its functional value is crucial and has to be taken into account. Improvement of accuracy during surgery results in better visual and functional outcomes simultaneously. Thus, a measurement tool to assess these two classifications is significant regarding the holistic approach in contemporary surgeries [23].

Since there is disagreement between individual studies regarding the impact of rhinoplasty on quality of life, this systematic review and meta-analysis aimed to analyze a larger population and establish whether there was a significant difference between pre- and post-treatment data in patients who underwent such a procedure. Moreover, there is a debate concerning the selection of the questionnaire that should be used in nasal reconstruction surgery and rhinoplasty. The Rhinoplasty Outcome Evaluation (ROE) questionnaire is well known and easy to use. It allows for the assessment of rhinoplasty-related satisfaction in patients. Therefore, we decided to conduct the present meta-analysis based on the results of this questionnaire that are available in the literature.

As presented in Table 1, the time at which the ROE was used to evaluate outcomes is different in each study. As we know, rhinoplasty is a surgery for which its final effects depend on the length of time from surgery. Because of that, it seems to be quite relevant that the group of studies has no common length of time from surgery to use as a control.

The reported values from the ROE in rhinoplasty patients varied between the studies [9,10,11,12,13,14,15,16]. This might be linked to the fact that each study included patients with different lengths of postoperative follow-up or different ages of patients in the included patient groups. The mean age of patients was 44.8 years in a study by Chang et al. and 36.3 years in a study by Rabaioli et al. [7,18]. Arima et al. showed that patients from younger age groups reported lower postoperative satisfaction scores than older patients [29]. Moreover, as there are no data concerning the gender of the participants, we were not able to divide groups into sex-based subgroups to discover potential sources of heterogeneity.

Regarding the selection for the meta-analysis, studies by Sasidran et al., Plath et al., Ozturk et al., Hudise et al., Avcu et al., Kook et al., and Kim et al. were not considered [30,31,32,33,34,35,36,37]. Ozturk et al. and Hudise et al. did not provide the standard deviation, and Plath et al. also included oncological patients, which might have influenced the results of the questionnaire [30,31,34,35]. Sasidran et al. provided two different data sets of post-treatment SD reporting. Arima et al. [38] followed patients who had rhinoplasty to correct the nose over a time period from 12 months to 8.4 years. In another paper by Arima et al. [29] the mean follow-up time after rhinoplasty was 55.9 months (ranging from 6 to 10 years) with the patients being divided into two subgroups according to the follow-up period, 6 to <60 months and ≥60 months, so it was also not included in the meta-analysis. Moreover, the ROE questionnaire was filled in by telephone contact. Similarly, the study of Sezgar et al. was not included in the meta-analysis as the values of the ROE scores pre-treatment as well as post-treatment are much lower than in the considered studies, which could be due to anthropometric hallmarks and cultural habits of the group of patients [37]. Avcu et al. considered numerous subgroups of patients, Kook et al. reported the results of the modified ROE questionnaire, and Kim et al. did not report the mean value of the ROE [33,36].

**Table 1:** Summary of the characteristics of the group of patients.

Publi+M18+A1:A1:M20	Number of Subjects	Quantity of Group, F	Quantity of Group, M	Age of Group Mean	Age of Group SD	Age of Group Range	ROE Score Pre-Treatment Mean	ROE Score Pre-Treatment SD	ROE Score Post-Treatment Mean	ROE Score Post-Treatment SD	Follow-up Mean or Exact [Months]
Rabaioli et al. [7]	131	78	53	36.33	14.12		21.24	3.88	58.59	5.83	3
Rabaioli et al. [7]	131	78	53	36.33	14.12		21.24	3.88	52.02	5.41	6
Kemal et al. [8]	55	27	28	29.5	9.1	18–57	46.36	9.28	78.85	11.7	6
Chang et al. [18]	28	17	11	37.29	13.06	20–62	43.3	12.54	67.56	19.75	6
Chang et al. [18]	26	17	9	44.85	13.47	19–71	40.7	14.16	58.98	14.13	6
Bulut 2017 et al. [11]	102	51	51	28.7	11.4		42.2	15.7	63.9	18.9	12
Moura et al. [9] m1	25	11	14	36.36	12.87		28.2	15.2	68.8	20.8	3
Moura et al. [9] m2	24	14	10	35.75	16.1		22.5	17	69.7	20	3
Mahato et al. [10]	117	50	67	28		18–50	26.5	6.95	75.21	9.58	3
Mahato et al. [10]	117	50	67	28			26.5	6.95	75.13	9.48	6
Mahato et al. [10]	117	50	67	28		18–50	26.5	6.95	76	8.36	12
Bayram et al. [12]	120	12	108	24		21–32	13.75	7.92	85	13.33	16
AlAwadh et al. [14]	21	16	5	25.9	5.56	17–37	27.58	11.88	82.33	10.29	12
Sabino et al. [15]	95	41	54	35.52	9.86	17–59	40.08	7.07	92.45	5.28	12
Suh et al. [16]	30	28	2	23.6	1.08	17–38	33.61	2.75	90.42	1.37	11.1
Sales et al. [39]	20	11	9	28.90			41.13	19.71	80	14.38	8.9
Barcagalia et al. [40]	21	5	16	40	7.6		20.23	7.37	80.75	6.24	5 years
Gökçe et al. [41]	90	58	32	27.4	6.5		30.5	4.65	72.5	2.7	6
Jahandideh et al. [42]	23	15	8	29.47	5.5		37.86	10.64	84.96	9.95	12
Jahandideh et al. [42] CT	26	19	7	30.88	7.07		42.62	9.81	90.22	6.65	12
Jahandideh et al. [42] C	25	17	8	29.24	2.01		40	13.39	83.99	8.89	12
Sazgar et al. [37]	23	18	5	32.2	4.3		6.36	3.69	17.27	4.67	12
Riedel et al. [43]	64	53	11	34.5	10.3		32.3	14.1	75.1	17.2	12
Haddady et al. [24]	60	52	8	26.6	8.5		51.27	10.54	79.6	9..67	6
Bulut 2015 et al. [21]	148	85	63	28.5	6.9	18–46	28.5	9	87.3	5	12
Cingi et al. [20]	191					18–57	19.77	7.99	76.2	17.46	12
Bulut 2018 et al. [13]	69	37	32				42.2	15.7	63.9	18.9	12

This study is not devoid of limitations. In particular, the studies were conducted in different countries. Moreover, some authors suggested that the ROE scale should be adapted to consider anthropometric hallmarks and cultural habits, as it is difficult to standardize a patient satisfaction scale if this depends on different attributes and cultural beliefs [44,45]. In addition, different techniques were used (e.g., rhinoplasty using silicone implants in a study by Rabaioli et al. and rhinoseptoplasty with EPIT in a study by Moura et al.) [46]. However, some reports did not confirm the influence of surgical approach on ROE scores, as in Bulut et al. [11]. There were different postoperative follow-up processes (from 3 months up to 24 months), but based on the literature this should not have an influence on the ROE score. However, the subgroup analysis conducted by us suggested that the different times of grading of postoperative follow-up processes impacted the overall meta-analysis. The groups of patients were not homogenous as different sets of criteria were used (e.g., Rabaioli et al. included patients with symptoms of body dysmorphic disorder) [7].

The ROE seems to be an appropriate tool with which to evaluate the satisfaction and final results of rhinoplasty despite the individual differences in results from human diversity. Currently, the non-Caucasian population constitutes an important group of patients interested in the subject of rhinoplasty. This heterogenous group consists of people of varying cultures, races, and ethnicities. Evaluation of these patients requires great understanding of these differences and the ability to define what their aesthetic ideals are and what will be needed for surgery. Nevertheless, the universal goal when treating non-Caucasian patients is the same—ensuring that patients achieve their requirements in the best possible fashion [47].

Rhinoplasty in patients of African descent requires a patient-specific approach, because it must be considered based on different norms than for Caucasian noses [48].

A very specific group seeking rhinoplasty is a subgroup of people with Asian noses, the philosophy of rhinoplasty specific to this group is augmentation rather than reduction in which the dorsum is raised and the nasal tip projection and definition are enhanced. The watchword today is “ethnic preservation” rather than “effacement” for the Asian patient. An ethnically sensitive standard for aesthetic rhinoplasty for the Asian nose has been proposed [49].

The ROE also seems to be a great measuring outcome tool in other groups of non-Caucasian origin. Mahmoudi S et al. used the Rhinoplasty Outcome Evaluation (ROE) to evaluate rhinoplasties among the Iranian population. They found the questionnaire to be easy to use, and they showed that it allows comprehensive assessment of rhinoplasty-related patient satisfaction. However, the normal values for this questionnaire are not known. They found the Iranian version of the ROE to be a valid instrument to assess results in that population [50,51]. Çelik M et al. had a very similar observation. They enrolled 30 participants who were native Turkish and underwent primary rhinoplasty. The control group consisted of 58 healthy volunteers. The reliability of the Rhinoplasty Outcomes Evaluation-T was analyzed according to its internal consistency and test-retest reproducibility. The Rhinoplasty Outcomes Evaluation-T seems to be a valid questionnaire to measure rhinoplasty outcomes among Turkish patients [52].

We must measure aspects of satisfaction in cosmetic procedures, although we already know that there are some problematic subgroups that we must consider.

Body dysmorphic disorder and borderline personality disorder occur in up to 15% of patients seeking aesthetic procedures. It has been shown that performing surgery on this group of patients may worsen their previous condition [52]. Psychosocial problems may motivate the demand for aesthetic rhinoplasty. Surgery in major cases improves the quality of life and self-esteem of patients. Nevertheless, those suffering from major depressive disorder, generalized anxiety disorder, or other conditions may seek aesthetic rhinoplasty as a solution [53]. In these subgroups, the ROE needs further validation.

We must consider, as well, that articles included in our meta-analysis were examining different patient groups. Some of the cases were revision cases, and some of them were big reconstruction cases (as shown in Table 2). So, in future investigations it would be beneficial to create a more specific groups, which can determine the range of surgeries and, that way, modify the potential outcomes. Some of them were primary cases with potentially better outcomes, and some of them were secondary cases that needed bigger grafting.

The results of this study demonstrated a significant difference between pre-treatment and post-treatment ROE results with a higher value of ROE score noted after treatment. This may suggest the impact of the rhinoplasty procedure on patients’ lives and should be a crucial argument, for example, for insurance companies in the insurance refund for the procedure.

When searching the literature to evaluate this topic, we noted that Arima et al. [38] found no difference in the quality of life between follow-up periods of 6 months and 10 years. However, in general, most articles revealed an improvement in quality of life. A meta-analysis showed different levels of QoL improvement in nasal obstruction [54]. Moreover, some authors observed that concomitant turbinate surgery had no influence on the nasal passage [55]. An increasing quantity of data showed a lower impact of septoplasty than had been believed before. Septoplasty (the surgical correction of a deviated nasal septum) is the most frequent laryngological procedure performed in adults. Nevertheless, published data are limited as regards to the strong level of evidence on the effectiveness of septoplasty. Therefore, trials are ongoing to try to measure the effectiveness of septoplasty with EBM gold standards [56]. Rhinoplasty is a more comprehensive procedure, so more aspects than just nasal congestion measurements have to be assessed. Hence, it seems that an improvement in the quality of life (in all its aspects) is the outcome that determines the success of cosmetic surgery. Patient-reported outcome measures (PROMs) constitute the tool to measure such areas in medicine. PROMs for clinical trials, effectiveness studies, and public health research have been defined as ‘‘any report coming directly from subjects without interpretation of the physician or others about how they function overall or feel in relation to a condition and its therapy’’ [57,58]. Therefore, we decided to conduct our systematic review using the ROE, which seems to be such a tool [50,59].

## 5. Conclusions

In summary, this study demonstrated a statistically significant difference between pre- and post-treatment ROE scores. The mean value of the pre-treatment ROE score was 33.50 with a CI of 29.46 to 37.53 (*p* < 0.001); for the post-treatment score it was 69.60 with a CI of 63.07 to 76.14 (t ≤ 6 months), and at t = 12 months it was 80.25 with a CI of 75.79 to 84.70 (*p* < 0.001). The mean difference between pre-treatment and post-treatment scores (t ≤ 6 months) was −36.31 with a CI of −40.93 to −31.69. The mean difference between pre-treatment and post-treatment scores for 6 m < t ≤ 12 m was −47.36 with a CI of −53.89 to −40.83. It is important to note that the received mean values of ROE scores after t = 12 m are comparable to the normative ROE scores, which are 73.1 ± 15.9. The ROE seems to be an appropriate tool to evaluate the satisfaction of patients after rhinoplasty; it is easy to carry out, and it is a valuable tool in clinical practice [60].

## Figures and Tables

**Table 2:** Summary of patient groups examined in meta-analysis studies.

Publication	Presenting Deformity/Technique Used
Rabaioli 2022	Rhinoseptoplasty—patients with symptoms of body dysmorphic disorder
Kemal 2022	Septorhinoplasty
Chang 2022	Xenograft implanting for rhinoplasty
Chang 2022	Rhinoplasty using silicone implants
Moura 2017	Primary septorhinoplasty, subgroups of non-deviated and deviated noses
Bulut 2018	Septorhinoplasty
Mahato 2021	Primary rhinoseptoplasty with partial inferior turbinectomy
Bayram 2018	Septorhinoplasty—closed and open approaches
Alawadh 2022	Late correction of traumatic nasal deformities
Sabino 2022	Modified spreader flap technique
Suh 2023	Rhinoplasty in caudal deviation of septum cases
Sales 2021	Revision rhinoplasty, managing alar retraction
Gökçe 2019	Rhinoplasty open approach
Bracaglia 2020	Revision rhinoplasty—tip supporting technique
Jahandideh 2020	Rhinoplasty open approach
Amer 2018	Rhinoplasty for nasal deformities in rhinoscleroma patients
Bulut 2017	Primary septorhinoplasty, subgroups of non-deviated and deviated noses
Riedel 2022	Revision rhinoplasty with cartilage grafting
Haddady 2021	Rhinoplasty open approach
Bulut 2015	Rhinoplasty open approach
Cingi 2013	Rhinoplasty open approach
